# Supplementary material for: Content-rich biological network constructed by mining PubMed abstracts
Source: BMC Bioinformatics. 2004 Oct 8;5:147. doi: 10.1186/1471-2105-5-147 (PMC528731; doi:10.1186/1471-2105-5-147)
Supplement: Additional File 2 — The original results of the above study (non-essential files are deleted to keep the file size under the limit set by BMC bioinformatics). [file 1471-2105-5-147-S2.bz2 › chilibotAdditionalFile2/dip30/459ID10488147E10477/html/CX3CL1_CX3CR1.html]

 


 **CX3CL1** and **CX3CR1** 
  
Found 71 abstracts in PubMed, retrieved 30.  
 

 What does Google say? 
 PDF only 
| .edu only 

---

**Interactive relationship** (e.g. stimulation, inhibition, etc)

**Neutral relationship**- CONCLUSION Our findings suggest that the interactions of  **CX3CL1**  and  **CX3CR1**  might contribute to the accumulation of  **CX3CR1**  T cells expressing type 1 cytokines and possessing cytotoxic granules in RA synovium.  Ref: 12428227 Arthritis Rheum, 2002
- The chemokine receptor  **CX3CR1**  is a proinflammatory leukocyte receptor specific for the chemokine fractalkine  [ **CX3CL1** ]  FKN or  **CX3CL1** .  Ref: 12697743 J Clin Invest, 2003
- Dual functions of fractalkine  [ **CX3CL1** ]  CX3C  [ **CX3CL1** ]  ligand 1 in trafficking of perforin granzyme B cytotoxic effector lymphocytes that are defined by  **CX3CR1**  expression.  Ref: 12055230 J Immunol, 2002
- BACKGROUND Fractalkine  [ **CX3CL1** ]  is a novel chemokine that mediates both firm adhesion of leukocytes to the endothelium via  **CX3CR1**  and leukocyte transmigration out of the bloodstream.  Ref: 12459434 Cardiovasc Pathol< MedlineTA>Cardiovasc Pathol, 2002
- Fractalkine  [ **CX3CL1** ]  is a neuronally expressed chemokine that acts through its G protein coupled receptor  **CX3CR1** , localized on microglial and immune cells.  Ref: 12059974 Eur J Neurosci, 2002
- The human hepatocarcinoma cell line HepG2 expressed fractalkine  [ **CX3CL1** ]  at the gene and protein level, and HepG2 conditioned medium was chemotactic for cells overexpressing  **CX3CR1** .  Ref: 12076860 J Hepatol, 2002
- **CX3CR1**  staining was seen in the coronary arteries of all cases, with specific localization to regions with fractalkine  [ **CX3CL1** ]  staining.  Ref: 12459434 Cardiovasc Pathol< MedlineTA>Cardiovasc Pathol, 2002
- In contrast to CD16 monocytes, CD16 monocytes expressed high  **CX3CR1**  and CXCR4 but low CCR2 and CD62L levels and underwent efficient transendo thelial migration in response to fractalkine  [ **CX3CL1** ]  FKN.  Ref: 12810688 J Exp Med, 2003
- BACKGROUND Fractalkine  [ **CX3CL1** ]   **CX3CL1** , a CX3C  [ **CX3CL1** ]  chemokine, is expressed in the vessel wall and mediates the firm adhesion and chemotaxis of leukocytes expressing its receptor,  **CX3CR1** .  Ref: 12600915 Circulation, 2003
- Decreased atherosclerosis in  **CX3CR1**  mice reveals a role for fractalkine  [ **CX3CL1** ]  in atherogenesis.  Ref: 12569158 J Clin Invest, 2003

**Non-interactive relationship** (e.g. studied together, co-existance, homology, etc.)

- We evaluated by in situ hybridization and immunohistochemistry the expression of fractalkine  [ **CX3CL1** ]  and  **CX3CR1**  in the rat brain, after a transient occlusion of the middle cerebral artery.  Ref: 12059974 Eur J Neurosci, 2002
- Transcripts for  **CX3CR1**  were detected in HepG2, and exposure of these cells to recombinant fractalkine  [ **CX3CL1** ]  induced cell migration.  Ref: 12076860 J Hepatol, 2002
- Consistent with this, intravenous injection of a single dose of recombinant IL 15 into C57BL 6 mice decreased steady state  **CX3CR1**  levels 24 hours after injection in freshly isolated PBMCs, splenocytes and bone marrow cells, and treatment of mouse PBMCs with IL 15 in vitro inhibited  **CX3CL1**  induced chemotaxis.  Ref: 12881312 BloodBlood, 2002
- To explore the relationship between TGF beta1 and  **CX3CR1**  in the CNS, the effects of TGF beta1 on  **CX3CR1**  mRNA, protein and fractalkine  [ **CX3CL1** ]  dependent stimulation of signal transduction cascades in primary cultures of rat microglia were examined.  Ref: 12446007 J Neuroimmunol, 2002
- METHOD Polyclonal rabbit antibodies were used to immunostain human fractalkine  [ **CX3CL1** ]  and  **CX3CR1**  to localize their presence in transverse sections of the proximal left anterior descending and or right coronary arteries.  Ref: 12459434 Cardiovasc Pathol< MedlineTA>Cardiovasc Pathol, 2002
- TGF beta1 increased steady state levels of  **CX3CR1**  mRNA, 125I fractalkine  [ **CX3CL1** ]  binding sites and blunted fractalkine  [ **CX3CL1** ]  stimulated ERK1 2 phosphorylation.  Ref: 12446007 J Neuroimmunol, 2002
- In this study, we characterized the presence of fractalkine  [ **CX3CL1** ]  and its receptor  **CX3CR1**  in human coronary arteries from normal, atherosclerotic, diabetic, and TVD settings.  Ref: 12459434 Cardiovasc Pathol< MedlineTA>Cardiovasc Pathol, 2002
- Fractalkine  [ **CX3CL1** ]  expression and the recruitment of  **CX3CR1**  cells in the prolonged mesangial proliferative glomerulonephritis.  Ref: 12028445 Kidney Int, 2002
- CONCLUSIONS Fractalkine  [ **CX3CL1** ]  expression and the recruitment of  **CX3CR1**  cells in glomeruli might play an important role in the development of the prolonged disease.  Ref: 12028445 Kidney Int, 2002
- In this study, we examined the role of  **CX3CL1**  fractalkine  [ **CX3CL1** ]  and its receptor CX3C  [ **CX3CL1** ]  chemokine receptor 1  [ **CX3CR1** ]   **CX3CR1**  in the T cell migration into RA synovium.  Ref: 12428227 Arthritis Rheum, 2002
- Both fractalkine  [ **CX3CL1** ]  and  **CX3CR1**  were expressed constitutively in neurons, microglia, and astrocytes.  Ref: 12850572 Brain Res, 2003
- CONCLUSIONS The  **CX3CR1**   **CX3CL1**  pathway seems to play a direct and critical role in monocyte recruitment and atherosclerotic lesion development in a mouse model of human atherosclerosis.  Ref: 12600915 Circulation, 2003
- CONCLUSION The distinctive staining patterns in native atherosclerosis, diabetes mellitus with atherosclerosis, and TVD indicate that the expression of fractalkine  [ **CX3CL1** ]  and  **CX3CR1**  may be important in the pathogenesis of these diseases.  Ref: 12459434 Cardiovasc Pathol< MedlineTA>Cardiovasc Pathol, 2003
- OBJECTIVE To investigate the expression of fractalkine  [ **CX3CL1** ]  FKN and its receptor  **CX3CR1**  in cardiac allografts and the effect of Cyclosporin A CsA.  Ref: 12783679 Zhonghua Wai Ke Za Zhi, 2003
- The aim of this study was to investigate the expression patterns of fractalkine  [ **CX3CL1** ]  and its receptor  **CX3CR1**  in normal human liver and in conditions of injury.  Ref: 12076860 J Hepatol, 2002
- TGF beta1 upregulates  **CX3CR1**  expression and inhibits fractalkine  [ **CX3CL1** ]  stimulated signaling in rat microglia.  Ref: 12446007 J Neuroimmunol, 2002
- Up regulated expression of fractalkine  [ **CX3CL1** ]  and its receptor  **CX3CR1**  during liver injury in humans.  Ref: 12076860 J Hepatol, 2002
- METHODS Using flow cytometry, immunohistochemistry, and reverse transcription polymerase chain reaction, we analyzed  **CX3CR1**  expression by peripheral blood and synovial T cells, and  **CX3CL1**  expression in synovium from patients with RA.  Ref: 12428227 Arthritis Rheum, 2002
- Fractalkine  [ **CX3CL1** ]  has been identified as a novel chemokine that exhibits cell adhesion and chemoattractive properties in the central nervous system CNS, and the fractalkine  [ **CX3CL1** ]  receptors,  **CX3CR1** , are also expressed in the CNS.  Ref: 12125082 J Neurosci Res, 2002
- Ad35K infected cell culture medium, containing recombinant 35K, potently reduced migration of CCR5 transfected 293 cells by 95% in response to the CC chemokine RANTES, but had no effect on cells transfected with the  **CX3CR1**  fractalkine  [ **CX3CL1** ]  receptor.  Ref: 12777060 Immunobiology, 2003
- RESULTS Both fractalkine  [ **CX3CL1** ]  and  **CX3CR1**  were up regulated during chronic injury, in areas of portal and lobular inflammation.  Ref: 12076860 J Hepatol, 2002
- In severe acute hepatitis, fractalkine  [ **CX3CL1** ]  and  **CX3CR1**  were expressed at high levels not only in areas of inflammation but also in regenerating epithelial cells within bile duct like structures, which showed co expression of fractalkine  [ **CX3CL1** ]  and cytokeratin 7 or  **CX3CR1** .  Ref: 12076860 J Hepatol, 2002
- Fractalkine  [ **CX3CL1** ]   **CX3CL1**  is a chemokine which is expressed predominantly in the central nervous system, being localized on neurons, while its receptor,  **CX3CR1** , is found on microglial cells.  Ref: 12725801 Methods, 2003
- Upon binding to the CX3C  [ **CX3CL1** ]  receptor one  **CX3CR1** , FKN induces adhesion, chemoattraction, and activation of leukocytes, including brain macrophages and microglia MP.  Ref: 12476352 J Neurovirol, 2002
- Expression of fractalkine  [ **CX3CL1** ]  and the number of the fractalkine  [ **CX3CL1** ]  receptor  **CX3CR1**  positive cells in the glomeruli in the prolonged model were significantly higher than those in the reversible model.  Ref: 12028445 Kidney Int, 2002
- Characterization of fractalkine  [ **CX3CL1** ]   **CX3CL1**  and  **CX3CR1**  in human coronary arteries with native atherosclerosis, diabetes mellitus, and transplant vascular disease.  Ref: 12459434 Cardiovasc Pathol< MedlineTA>Cardiovasc Pathol, 2002
- Then, the localization of fractalkine  [ **CX3CL1** ]  and the characteristics of  **CX3CR1**  cells were analyzed in glomeruli.  Ref: 12028445 Kidney Int, 2002
- Although the treatment did not ameliorate the morphological finding at two weeks, decreased expression of fractalkine  [ **CX3CL1** ]  and  **CX3CR1**  were already detected at two weeks in rats treated with candesartan.  Ref: 12028445 Kidney Int, 2002
- **CX3CL1** , the unique ligand of  **CX3CR1** , was expressed by endothelial cells and synoviocytes in RA synovium, but not in osteoarthritis synovium.  Ref: 12428227 Arthritis Rheum, 2002
- BACKGROUND  **CX3CL1**  fractalkine  [ **CX3CL1** ]  is a membrane bound chemokine that can function as an adhesion molecule for cells expressing the receptor  **CX3CR1** .  Ref: 12110009 Kidney Int, 2002
- In the present study, we examined the production of fractalkine  [ **CX3CL1** ]  and the expression of its receptor,  **CX3CR1**  in murine glial and neuronal cell in vitro, and investigated its neuroprotective functions.  Ref: 12850572 Brain Res, 2003
- In two examples, we showed that chemokines influence notably the cellular immune functions, such as CD8 cytotoxicity Rantes CCR3 and gamma interferon production fractalkine  [ **CX3CL1** ]   **CX3CR1** .  Ref: 12134637 J Soc Biol, 2002
- Expression of the fractalkine  [ **CX3CL1** ]  receptor  **CX3CR1**  in human kidney diseases.  Ref: 12110009 Kidney Int, 2002
- Transient transfections with dnIkappaB alpha, dnIkappaB beta, dnIKK gamma, kdNIK, and kdIKK beta or pretreatment with wortmannin, Akt inhibitor, PDTC, or MG 132 significantly attenuated TNF alpha induced fractalkine  [ **CX3CL1** ]  and  **CX3CR1**  expression.  Ref: 12729461 Biochem J< MedlineTA>Biochem J, 2002
- We have previously shown that reduced expression of the fractalkine  [ **CX3CL1** ]  receptor,  **CX3CR1** , is correlated with rapid HIV disease progression and with reduced susceptibility to acute coronary events.  Ref: 12234253 Biochem J, 2002
- Expression of fractalkine  [ **CX3CL1** ]  and its receptor,  **CX3CR1** , in response to ischaemia reperfusion brain injury in the rat.  Ref: 12059974 Eur J Neurosci, 2002
- Interactions between fractalkine  [ **CX3CL1** ]  and  **CX3CR1**  can mediate not only chemotaxis but also cell adhesion in the absence of substrates for other adhesion molecules.  Ref: 12806431 Drug News Perspect, 2001
- Fractalkine  [ **CX3CL1** ]  receptor  **CX3CR1**  is expressed on natural killer NK cells, monocytes and some portions of CD8 T cells.  Ref: 12806431 Drug News Perspect, 2001
- OBJECTIVESWe have recently reported that the polymorphism of the fractalkine  [ **CX3CL1** ]  receptor,  **CX3CR1** , provides a new marker for prognosis in HIV disease.  Ref: 12799549 AIDS, 2003
- Studies using a static model of adhesion demonstrated  **CX3CR1**  fractalkine  [ **CX3CL1** ]  interactions accounted for 26% of monocytic THP 1 cell and 17% of peripheral blood natural killer cell adhesion to tubular epithelial cells, suggesting that fractalkine  [ **CX3CL1** ]  may have a functional role in leucocyte adhesion and retention, at selected tubular sites in acute renal inflammation.  Ref: 12100035 Clin Exp Immunol, 2002
- Pretreatment with pertussis toxin or anti  **CX3CR1**  neutralizing antibodies attenuated TNF alpha induced fractalkine  [ **CX3CL1** ]  induction indicating that fractalkine  [ **CX3CL1** ]  autoregulation plays a role in TNF alpha induced sustained fractalkine  [ **CX3CL1** ]  expression.  Ref: 12729461 Biochem J< MedlineTA>Biochem J, 2002
- Fractalkine  [ **CX3CL1** ]  CX3C  [ **CX3CL1** ]  ligand 1 and its receptor  **CX3CR1**  are known to mediate both cell adhesion and cell migration.  Ref: 12055230 J Immunol, 2002
- TNF alpha activated NF gammaB and induced fractalkine  [ **CX3CL1** ]  and  **CX3CR1**  expression in a time dependent manner in rat aortic smooth muscle cells.  Ref: 12729461 Biochem J< MedlineTA>Biochem J, 2002
